# Case report: Clinical profile, molecular genetics, and neuroimaging findings presenting in a patient with Kearns-Sayre syndrome associated with inherited thrombophilia

**DOI:** 10.3389/fneur.2023.1320757

**Published:** 2024-01-05

**Authors:** Anca Elena Gogu, Dragos Catalin Jianu, Florina Parv, Andrei Gheorghe Marius Motoc, Any Axelerad, Alina Zorina Stuparu, Andreea Alexandra Gogu

**Affiliations:** ^1^Department of Neurology, “Victor Babeş” University of Medicine and Pharmacy, Timișoara, Romania; ^2^Centre for Cognitive Research in Neuropsychiatric Pathology (Neuropsy-Cog), Faculty of Medicine, “Victor Babeş” University of Medicine and Pharmacy, Timișoara, Romania; ^3^Department of Cardiology, “Victor Babeş” University of Medicine and Pharmacy, Timișoara, Romania; ^4^Department of Anatomy and Embryology, “Victor Babeş” University of Medicine and Pharmacy, Timișoara, Romania; ^5^Department of Neurology, General Medicine Faculty, “Ovidius” University, Constanța, Romania; ^6^Medicine Faculty, “Victor Babeş” University of Medicine and Pharmacy, Timișoara, Romania

**Keywords:** Kearns-Sayre syndrome (KSS), inherited thrombophilia, heart conduction block, brain magnetic resonance imaging, genetic tests

## Abstract

**Background:**

Kearns-Sayre syndrome (KSS) is classified as one of the mitochondrial DNA (mtDNA) deletion syndromes with multisystemic involvement. Additionally, the negative prognosis is associated with inherited thrombophilia, which includes the presence of homozygous Factor V G1691A Leiden mutation, MTHFR gene polymorphisms C677T and A1298C, and PAI-1 675 homozygous genotype 5G/5G.

**Case presentation:**

This case report presents a 48-year-old man with chronic progressive external ophthalmoplegia, bilateral ptosis, cerebellar ataxia, cardiovascular signs (syncope, dilated cardiomyopathy, and cardiac arrest) with electrocardiographic abnormalities (first-degree atrioventricular block and major right bundle branch block), endocrine dysfunction (short stature, growth hormone insufficiency, primary gonadal insufficiency, hypothyroidism, and secondary hyperparathyroidism), molecular genetic tests (MT-TL2 gene), and abnormal MRI brain images, thus leading to the diagnosis of KSS. The patient came back 4 weeks after the diagnosis to the emergency department with massive bilateral pulmonary embolism with syncope at onset, acute cardiorespiratory failure, deep left femoral-popliteal vein thrombophlebitis, and altered neurological status. In the intensive care unit, he received mechanical ventilation through intubation. Significant improvement was seen after 2 weeks. The patient tested positive for inherited thrombophilia and was discharged in stable conditions on a new treatment with Rivaroxaban 20 mg/day. At 6 months of follow-up, ECG-Holter monitoring and MRI brain images remained unchanged. However, after 3 months, the patient died suddenly while sleeping at home.

**Conclusion:**

The genetic tests performed on KSS patients should also include those for inherited thrombophilia. By detecting these mutations, we can prevent major complications such as cerebral venous sinus thrombosis, coronary accidents, or sudden death.

## 1 Introduction

Kearns-Sayre syndrome (KSS), initially reported in 1958 at the Mayo Clinic (1, 2), is an uncommon mitochondrial myopathy. It is characterized by a greater severity compared to chronic progressive external ophthalmoplegia (CPEO), encompassing multiple systems in its manifestation. There are three cardinal clinical features of KSS: onset prior to 20 years of age, pigmentary retinopathy, and progressive external ophthalmoplegia (3, 4). Furthermore, the diagnosis of KSS requires the presence of at least one of the features, including cardiac conduction abnormalities, cerebellar ataxia, cerebrospinal fluid (CSF) protein level above 100 mg/dl, short stature, endocrine abnormalities, and cognitive decline (5).

Inherited thrombophilia genetic risk factors include factor V G1691A-Leiden mutation, prothrombin G20210A mutation, methylenetetrahydrofolate reductase (MTHFR) gene C677T and A1298C mutation, and plasminogen-activator inhibitor PAI-1 675 5G/5G mutation, correlated with cardiovascular risk factors, which represents an aggravating status for patients with KSS due to the risk of pulmonary embolism, deep venous thrombosis, or cerebral venous sinus thrombosis (CVST) (6, 7).

This article describes a case of Kearns-Sayre syndrome without a family history of KSS or other mitochondrial disorders in which inherited thrombophilia was detected, complicated with pulmonary embolism and deep venous thrombosis, leading to the patient's death. The patient was assessed by a multidisciplinary team (neurologist, cardiologist, endocrinologist, neuro-ophthalmologist, and neuro-radiologist) to establish a clinical diagnosis of KSS.

## 2 Case presentation

A 48-year-old male patient presented to the emergency department with headache, dizziness, unstable gait, limb weakness, accentuation of eyelid ptosis, external ophthalmoplegia, and sinus tachycardia. The disease manifested in the patient at the age of 16, initially presenting with a small stature and symptoms related to the extraocular muscles. As the disease progressed, the patient experienced a gradual onset of additional clinical characteristics such as heart conduction issues, endocrine dysfunction, and a slight cognitive decline.

**Figure 1:**
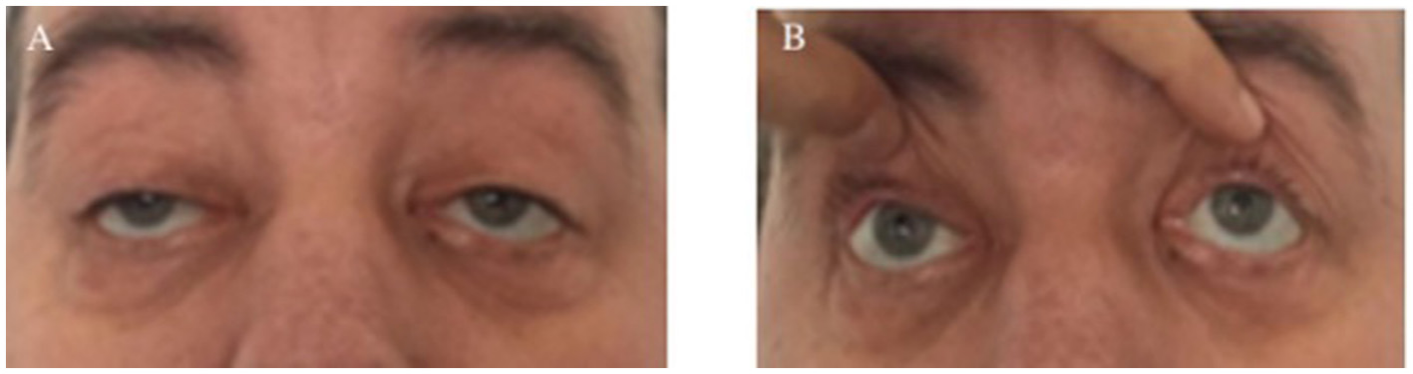
Clinical ophthalmologic symptoms. Bilateral ptosis **(A)** and external ophthalmoplegia with paralysis of vertical movements **(B)**.

The diagnosis of KSS was supported by identifying relevant clinical characteristics that indicated an abnormality in the patient's ophthalmologic system: bilateral ptosis, diplopia, chronic progressive external ophthalmoplegia, and a moderate decrease in visual acuity (Figure 1A). Considering that the patient's ophthalmoplegia did not match a specific group of cranial nerve palsies (oculomotor nerve palsy, fourth nerve palsy, and sixth nerve palsy), the likelihood for myopathies was even higher. Paralysis of the vertical movements of the eyeballs was observed (Figure 1B). No retinitis pigmentosa was discovered during the fundus examination performed by the ophthalmologist, but retinal angiosclerosis has been described.

Following ophthalmologic observations, the neurological examination highlights mild generalized muscle weakness, cerebellar ataxia, cerebellar tremor, and a slight cognitive impairment (Mini-Mental State Examination score of 22 points).

The patient had endocrinological symptoms and signs that preceded the neurological manifestations, including short stature (154 cm) caused by growth hormone insufficiency, delayed puberty, primary hypogonadism, hypothyroidism, secondary hyperparathyroidism, gynecomastia, frontal baldness, and brachydactily. The patient had no children.

**Figure 2:**
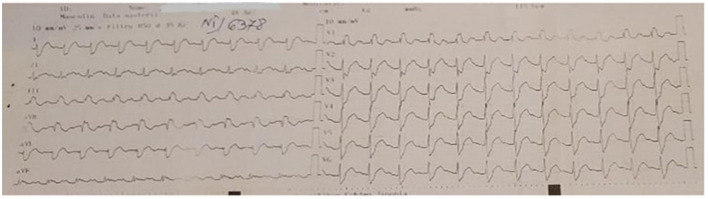
ECG–first-degree atrioventricular block, major right bundle branch block, and sinus tachycardia.

Cardiac involvement is common in disorders characterized by mitochondrial dysfunction due to their impact on organs that rely heavily on energy (8). Following the confirmation of the diagnosis, the patient underwent a series of medical tests, including 12-lead surface electrocardiography (ECG), transthoracic echocardiography, and 24-h Holter monitoring. Conduction abnormalities were seen, including first-degree atrioventricular block (AVB), significant right bundle branch block (RBBB), and sinus tachycardia (Figure 2). The echocardiogram revealed mild anterior mitral valve prolapses (MVP) and type I diastolic and systolic LV dysfunction (LVEF = 40%).

Given the presence of many clinical features suggesting KSS in this patient, we conducted a comprehensive analysis of biochemical and genetic tests related to cardiovascular risk factors. The lab testing demonstrated WBC of 12,460/μl (NV 4,000–9,500/μl), hemoglobin of 17.9 g/dl (NV 13.6–17.2 g/dl), hematocrit of 53.6% (NV 39–51%), blood glucose of 101 mg/dl (NV 74–106 mg/dl), aspartate aminotransferase of 58 U/L (NV 15–37 U/L), alanine aminotransferase of 71 U/L (NV 30–65 U/L), lactic dehydrogenase of 356 U/L (NV 85–227 U/L), creatinine kinase of 308 U/L (NV 39–308 U/L), and creatinine kinase-MB of 42 U/L (NV 7–25 U/L). The serum ionogram revealed ionic calcium of 3.95 mg/dl (NV 4.2–5.2 mg/dl), magnesium of 2.2 mg/dl (NV 1.8–2.4 mg/dl), potassium of 5.5 mmol/L (NV 3.5–5.1 mmol/L), and sodium of 149 mmol/L (NV 136–145 mmol/L).

Endocrinological analyses showed the following values: growth hormone (hGH) < 0.050 ng/ml (NV 0.4–10 ng/ml) and the thyroid hormone levels were FT3 of 3.35 pmol/L (NV 3.54–6.47 pmol/L), FT4 of 15.19 pmol/L (NV 11.48–22.70 pmol/L), TSH of 0, 150 mIU/ml (NV 0.55–4.78 mIU/ml), anti-TG of 24.6 U/ml (NV 0–60 U/ml), and anti-TPO of 31.9 U/ml (NV 0–60 U/ml). Intact PTH was 61.8 pg/ml (NV 7.5–53.5 pg/ml) with a low level of ionic calcium (3.95 mg/dl). Gonadotropic hormones were testosterone of 182.76 ng/dl (NV 241–827 ng/dl), FSH of 22.56 mIU/mL (NV 1.4–18.1 mIU/mL), and LH of 24.39 mIU/mL (NV 1.5–9.3 mIU/mL). Serum cortisol was normal (15.16 μg/dl). CSF indicated a high protein level of 91 mg/dl (NV 0.12–0.60 mg/dl) and a high glucose level of 131 mg/dl (NV 40–70 mg/dl) in the absence of white blood cells during a lumbar puncture procedure. For the differential diagnosis of myasthenia gravis, we tested anti-acetylcholine receptor antibodies, which were normal (AChR < 0.2 nmol/L).

Performing next-generation sequencing (NGS) on mitochondrial DNA extracted from peripheral blood leukocyte samples is the primary diagnostic method for identifying deletions in situations where KSS is clinically suspected (5, 9). We requested the testing for sequence analysis of the MT-TL2 gene; this test was developed and its performance was validated by CENTOGENE AG (Rostock, Germany) for clinical purposes. The blood sample was processed by enriching targeted sequences, and sequencing was done using NGS Technologies.

We did not detect any clear pathogenic variant in the MT-TL2 gene sequencing, including the variant m.12315G>A, which has been reported to be associated with KSS. However, it should be noted that the variants encoded in the mitochondrial genome may not be detected in blood if the percentage of heteroplasmy is low (typically < 15% heteroplasmy) (10). In this situation, it is recommended to test a sample from an affected tissue, but the patient did not agree to the muscle biopsy.

Electromyography (EMG) showed a myogenic pathway with small amplitude and short-duration motor unit potentials with an early recruitment pattern (bilateral frontal and deltoid muscles). Electroencephalography (EEG) revealed a generalized theta background pattern; the lesion-type route was maintained even when activated by hyperventilation or intermittent light stimulation (6–12 Hz).

**Figure 3:**
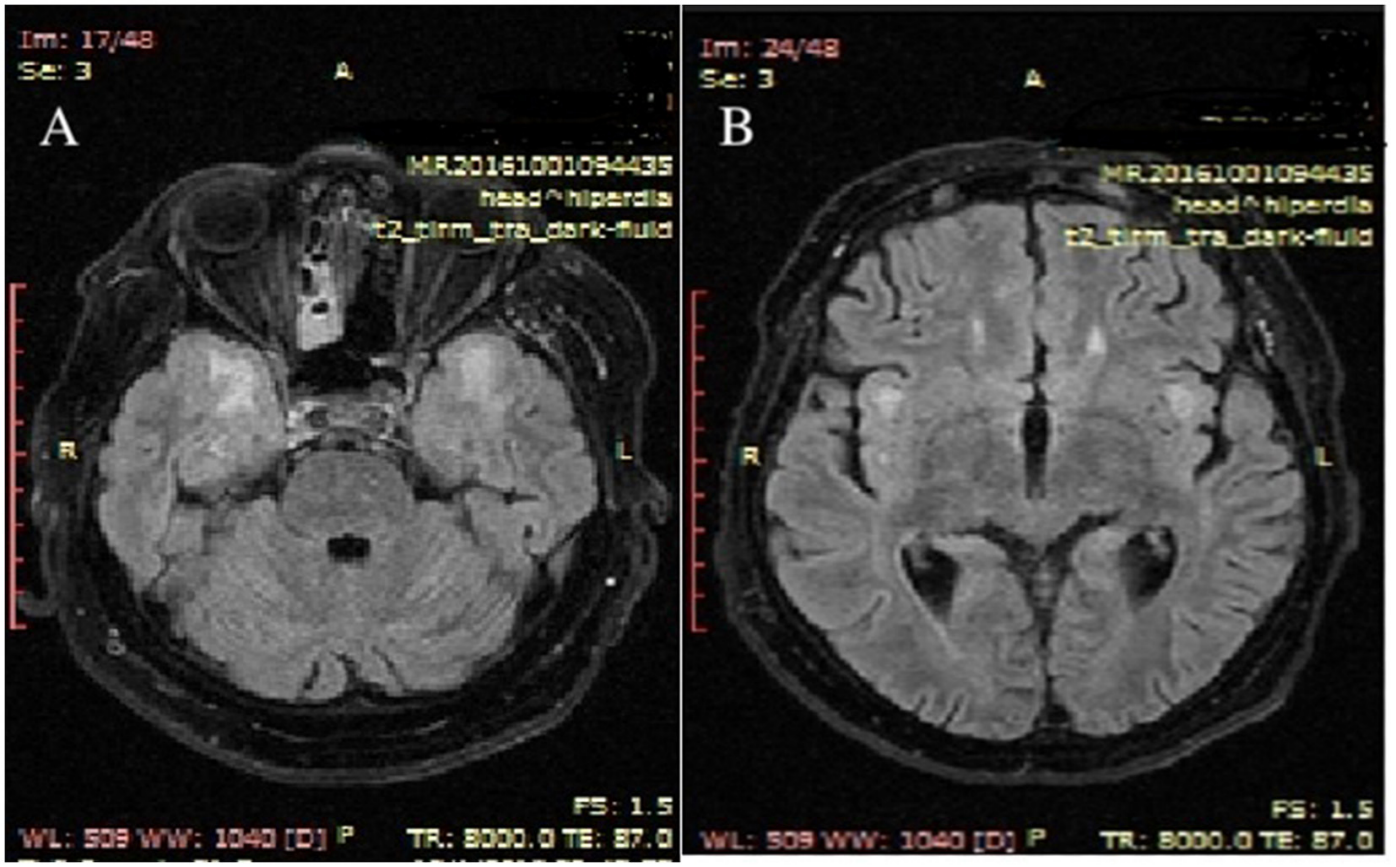
Upon admission to the hospital, MRI brain scans with contrast revealed T2 axial hypersignal in the subcortical white matter of both temporal lobes **(A)** and in the bilateral insula of the Reil lobes **(B)**. MRI stands for magnetic resonance imaging.

Upon admission to our clinic, brain magnetic resonance imaging (MRI) was performed with and without contrast. The results revealed symmetrical high-T2 and Flair signals in the cerebral white matter of both temporal lobes and in the bilateral insula of the Reil lobes (Figure 3).

**Figure 4:**
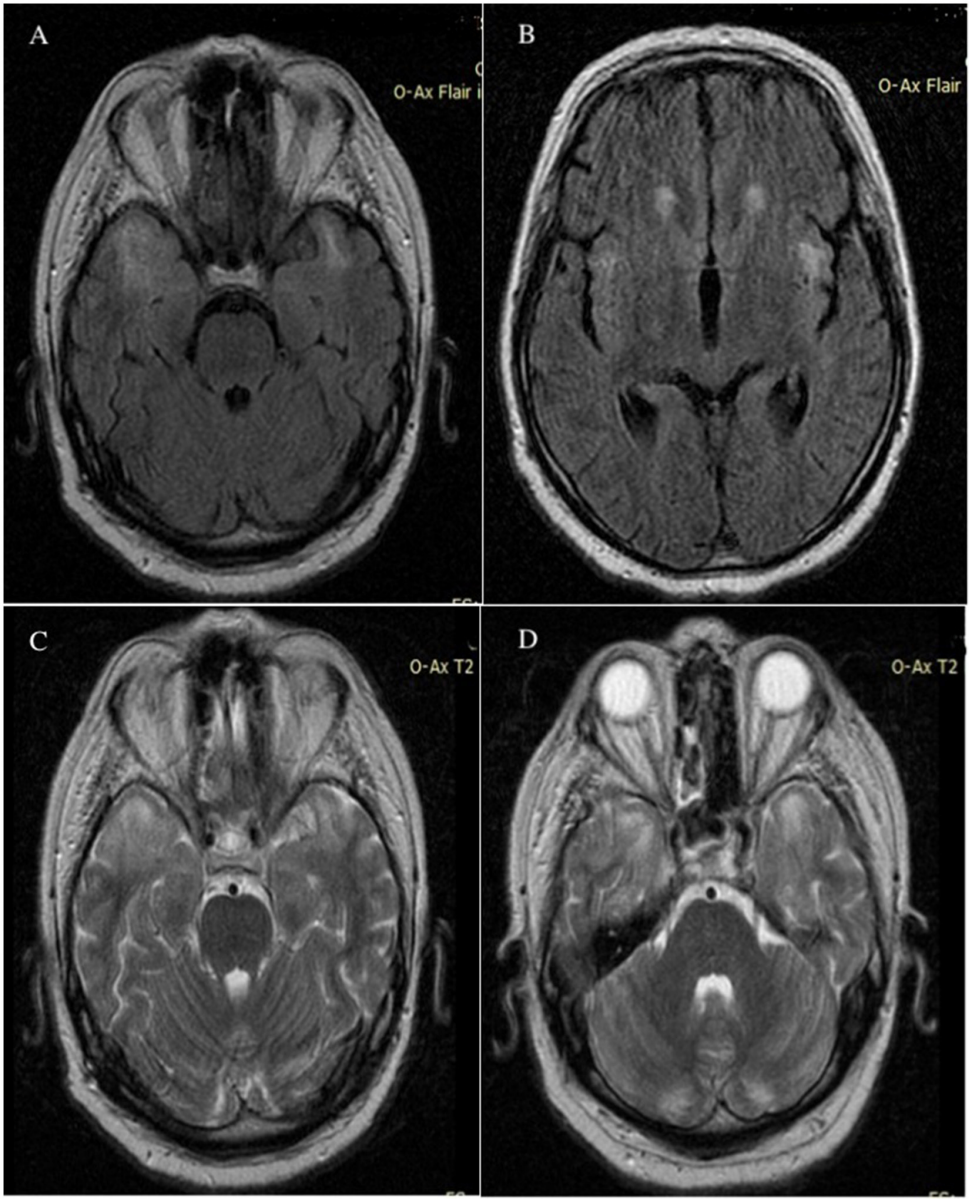
MRI axial Flair FSE images without contrast demonstrated abnormally high signals in the bilateral temporal lobes **(A)** and in the bilateral insula of the Reil lobes **(B)** MRI T2 axial images without contrast showed hypersignal in bilateral temporal lobes **(C)** and cerebellar white matter **(D)**; imaging appearance was unchanged after 6 months. MRI, magnetic resonance imaging; Flair, fluid attenuated inversion recovery; FSE, fast spin echo.

The appearance of brain lesions did not change; the patient underwent several brain MRIs in the year in which he was supervised. Unusual high-T2 signals were identified in the white matter of the cerebellum. Figure 4 shows the presence of cerebellar atrophy.

The patient was discharged in stable condition with the recommendation of cardiological monitoring. There is now no known remedy for this uncommon condition, and all available therapies only provide support. It was advised to undergo hormone replacement therapy and take Coenzyme Q10 (CoQ10). The patient presented at the emergency department 1 month later with a severe case of bilateral pulmonary embolism, accompanied by syncope at the onset, acute cardiorespiratory failure, proximal deep vein thrombosis in the left limb (the femoral-popliteal veins), and altered neurological status characterized by a depressed level of consciousness and extreme agitation. He received intubation and mechanical ventilation in the critical care unit.

The patient's laboratory results showed WBC of 10,800/μl, hemoglobin of 16.1 g/dl, hematocrit of 48.6%, platelets of 263,000/μl, blood glucose of 128 mg/dl, aspartate aminotransferase of 6,570 U/L, alanine aminotransferase of 5,735 U/L, lactic dehydrogenase of 4,736 U/L, creatinine kinase of 535 U/L, creatinine kinase-MB of 48 U/L, cholinesterase of 14,428 U/L (NV 5,320–12,920 U/L), and lactic acid of 3.2 mmol/L (NV 0.5–2.2 mmol/L). Serum inflammatory workup showed elevated hsCRP of 71.98 mg/L (NV 0–10 mg/L) and fibrinogen of 599 mg/L (NV 200–393 mg/L). The hepatitis panel (AcHBs, aHCV, and HAV Ig M) was negative. The hormone levels were FT3 of 2.55 pmol/L, FT4 of 15.08 pmol/L, TSH of 1,003 mIU/ml, and anti-TPO of 45.3 U/L; FSH of 2.76 mIU/ml, LH of 3.70 mIU/ml, and testosterone of 66.80 ng/dl. Coagulation tests were prothrombin time of 16.0 s (NV 9.4–12.5 s), APTT of 74.1 s (NV 25.1–36.5 s), and INR of 1.43 (NV 0.80–1.07).

Chest-computed tomography (chest-CT) revealed massive bilateral pulmonary embolism with multifocal infiltrates. The echocardiogram showed dilation of the right cardiac chambers and moderate secondary pulmonary hypertension. The venous Doppler ultrasonography of the lower extremities detected recent total thrombosis in the left femoral and popliteal veins. The patient was tested for inherited thrombophilia, obtaining the following genetic mutations: homozygous Factor V G1691A Leiden, MTHFR gene polymorphism C677T and A1298C, and PAI-1 675 homozygous genotype 5G/5G. The patient did not present a prothrombin G20210A mutation or protein C, protein S, or antithrombin deficiencies. In individuals with Kearns-Sayre syndrome, the presence of inherited thrombophilia, together with pre-existing cardiovascular disease, is indicative of a poor prognosis.

Significant improvement was seen after 2 weeks, and the patient returned home in stable conditions on a novel treatment with an oral anticoagulant drug (Rivaroxaban 20 mg/day). At 6 months of follow-up, ECG-Holter monitoring and MRI brain images remain unchanged. After 3 months, the patient died suddenly while sleeping at home.

## 3 Discussion

This is an exceptional instance where a patient has Kearns-Sayre syndrome (KSS) together with inherited thrombophilia, namely, the homozygous Factor V G1691A Leiden mutation, MTHFR gene polymorphisms C677T and A1298C, and PAI-1 homozygous genotype 5G/5G. Unfortunately, this combination of conditions leads to a catastrophic prognosis.

KSS is an uncommon mitochondrial disorder characterized by both systemic and ocular symptoms. These symptoms include chronic progressive external ophthalmoplegia (CPEO), pigmentary retinopathy, and a beginning of symptoms before the age of 20. Patients who have chronic progressive external ophthalmoplegia (CPEO) who satisfy some, but not all the criteria for KSS are referred to as having “KSS minus” or “CPEO plus” (1, 11).

The patient in our case report did not have a familial predisposition to KSS or any other mitochondrial illnesses. Our findings indicate that the patient had symptoms related to ophthalmology, neurology, cardiology, and endocrinology. This result aligns with the diagnosis of KSS.

The condition manifested in the second decade of life, with the first symptom being unilateral ptosis, which subsequently developed into bilateral ptosis and external ophthalmoplegia. We used a serial examination of old photos to establish the chronic, progressive nature of this disorder. As a particularity of this case, we did not find pigmentary retinopathy, and a dilated fundus examination revealed retinal angiosclerosis. The patient had considerably diminished visual acuity and was presenting with retinopathy.

Additionally, our investigation highlighted the presence of cardiovascular involvement. Yu et al. reported a study including 19 patients with KSS diagnosed with conduction defects that were aggravating but not constant, gradually deteriorating from bundle branch blockage to complete atrio-ventricular block (14). Other reports also demonstrated that the primary cause of sudden death in KSS patients was a complete atrio-ventricular block, and the incidence of sudden cardiac death was 20% as reported. The most common type of ventricular arrhythmia reported in KSS patients by others was bradycardia-related polymorphic ventricular tachycardia (14, 15). We identified conduction abnormalities characterized by first-degree atrioventricular block (AVB), major right bundle branch block (RBBB), and sinus tachycardia. The criteria for permanent pacemaker/implanted cardioverter defibrillator (PPM/ICD) insertion include high-grade heart block, bradycardia, a combination of heart block and bradycardia, and broadening of the QRS complex (1). Given the circumstances, the patient did not meet the criteria for PPM/ICD devices. Throughout a span of 1 year, the patient had ECG-Holter monitoring, which revealed no more abnormal heart rhythms. Prior research on progressive heart block and sudden cardiac death in individuals with KSS recommends considering regular preventive installation of a pacemaker (PPM) or implantable cardioverter-defibrillator (ICD). It also suggests having a low threshold to perform electrophysiological testing rather than doing formal electrophysiological testing in these patients, with a preference for a lower threshold (1, 12–14).

KSS has been associated with several endocrine disorders. Khambatta et al. reported a case series of 35 adults and children with KSS. They identified patients with short stature, delayed puberty, diabetes mellitus, and hypothyroidism. However, hyperaldosteronism and hypoparathyroidism, which have been reported in previous studies, were not observed in their study (1). Endocrine disturbances are frequently reported in KSS, similar to those in our patient, including short stature and brachydactyly from low growth hormone, delayed puberty, primary hypogonadism, frontal baldness, and gynecomastia caused by gonadotropic insufficiency and hypothyroidism. Usually, a patient with KSS has primary hypoparathyroidism, but in our case, the patient had been reported to have secondary hyperparathyroidism with an increased level of PTH and a low level of ionic calcium. Hypocalcemia associated with elevated PTH values in a patient with normal renal function suggests resistance to PTH actions.

Throughout the progression of the illness, the individual had a progressive onset of other neurological symptoms, including cerebellar ataxia, cerebellar tremor, muscular weakness in the limbs and shoulders, and a decline in cognitive abilities. The results of the brain magnetic resonance imaging (MRI), both with and without contrast, showed abnormalities. In a previous study conducted by other scholars, it was shown that the subcortical white matter had a 90.9% involvement rate, the basal ganglia and brainstem had a 63.6% involvement rate each, the thalamus had a 54.5% involvement rate, and the cerebellum had a 25.0% involvement rate (15, 16). The patient exhibited cerebral involvement, namely in the bilateral temporal lobes and bilateral insula of the Reil lobes, as well as cerebellar white matter involvement. However, there was no involvement of the brainstem. There was evidence of cerebellar atrophy. Subsequent investigations should include extended clinical and MRI monitoring to more accurately monitor the progression of brain MRI abnormalities and their correlation with clinical characteristics (15).

Prior published case series have included individuals diagnosed with KSS. Yamashita et al. reported on a group of 136 individuals who had mtDNA deletions. However, after using the diagnostic criteria established by Roland et al., they discovered that 24% of these patients fulfilled the criteria for Kearns-Sayre syndrome (KSS) (4, 17). Khambatta et al. conducted a study on 35 individuals with KSS. They relied on the diagnosis made by the treating physician instead of retrospectively applying criteria based on positive genetic test results (1). The patients exhibited more heterogeneity, including cases with onset occurring beyond the age of 20 years and lacking both progressive external ophthalmoplegia (PEO) and pigmentary retinopathy. Our investigation has constraints. No pathogenic variant was identified in the MT-TL2 gene sequencing. Additionally, due to logistical constraints, additional genetic testing could not be performed since it is only available in another country. If patients with a high likelihood of having KSS based on their physical characteristics have negative blood tests for KSS, it is recommended to sequence their muscle mtDNA. This is because tissue-specific mutations can sometimes be overlooked, and low levels of heteroplasmy in the blood can result in false-negative outcomes (5). Regrettably, the patient declined to have the muscle biopsy.

Since the patient returned to the emergency department with massive bilateral pulmonary embolism with syncope at onset, cardiorespiratory acute failure, proximal deep vein thrombosis, and altered neurological status without atrial fibrillation, we did tests for inherited thrombophilia, obtaining the following genetic mutations: homozygous Factor V G1691A Leiden, MTHFR gene polymorphisms C677T and A1298C, and PAI-1 675 homozygous genotype 5G/5G. A differential diagnosis was made with cerebral venous sinus thrombosis. The diagnostic assessment using magnetic resonance imaging (MRI) and magnetic resonance venography (MRV-2D TOF) disproved the diagnosis of cerebral venous sinus thrombosis (CVST).

A study conducted by Tripathi et al. (18) examined a population in North India and found a correlation between the MTHFR C677T gene polymorphism, elevated plasma homocysteine levels, and coronary artery diseases. Previous literature studies have shown the significance of the MTHFR C677T polymorphism in the development of ischemic stroke and cerebral venous sinus thrombosis (6, 7, 19, 20). MTHFR deficiency is an inherited disorder that affects the way the brain processes folate. In 2004, Ramaerkers et al. coined the term cerebral folate deficiency (CFD) to include any neuropsychiatric or neurodevelopmental diseases that are linked to low MTHFR concentration in cerebrospinal fluid (CSF), despite normal levels of folate, vitamin B12, and homocysteine outside the nervous system (21, 22). Mitochondrial abnormalities related to mutations in nuclear- or mitochondrial-encoded DNA are a significant cause of CFD syndrome. These mutations are responsible for mitochondrial encephalopathies, KSS, and Alper's illness. MTHFR deficiency is a genetic condition that causes elevated levels of homocysteine, leading to many symptoms, including developmental delay, eye issues, thrombosis, and osteoporosis (23). The presence of either homozygous (C677T) mutations or compound heterozygous (C677T and A1298C) mutations is anticipated to result in reduced MTHFR enzyme activity, which may present a variety of clinical neurological symptoms similar to those seen in infantile CFD (24, 25). In our patient diagnosed with KSS, it would have been necessary to dose folate in the CSF. MTHFR gene polymorphisms C677T and A1298C did not mean that the patient had cerebral folate deficiency, but determination of the folate level in the CSF would have been important to institute treatment with folic acid. In our patient diagnosed with KSS and having both heterozygous C677T and A1298C mutations, the presence of a cerebral folate deficit may play a role in the development of leukoencephalopathy and cognitive symptoms. Additionally, this deficiency significantly increases the likelihood of experiencing venous and arterial thrombosis. In addition to quantifying the proteins in the cerebrospinal fluid by lumbar puncture, we firmly believe that it is essential to determine the concentration of folic acid as early as possible in the evolution of the disease.

## 4 Conclusion

Patients with high suspicion of KSS must be examined by clinicians from a neurological, cardiological, endocrinological, and ophthalmological point of view. Specific medical tests may assist in the diagnosis, including genetic tests for mitochondrial DNA deletions, muscle biopsy, spinal tap (to assess the protein and folic acid in the cerebrospinal fluid), blood tests, EKG and echocardiogram, MRI of the brain, and screening for endocrinological disorders (dosing of hormones, especially thyroid, gonadotropins, and growth hormone).

This presentation in the patient with KSS associated with inherited thrombophilia is a coincidence discovered following thromboembolic complications that appeared with a reserved prognosis. We consider that genetic testing for inherited thrombophilia in KSS patients with pulmonary thromboembolism, deep vein thrombosis, or cerebral venous sinus thrombosis is necessary, including the following mutations: MTHFR gene polymorphism C677T and A1298C, PAI-1 675 homozygous genotype, Factor V G1691A Leiden, prothrombin G20210A mutation, protein C, protein S, and antithrombin deficiencies. The mutations in the MTHFR and PAI-1 genes were not clinically important alone, but when found with Factor V Leiden, they could contribute to the risk of thromboembolism. By detecting these mutations, we can prevent major complications that can have a fatal prognosis, such as cerebral venous sinus thrombosis, ischemic stroke, coronary accidents, or sudden death. Therefore, certain treatments should include oral anticoagulant drugs, folic acid, hormone replacement, and a pacemaker for heart rhythm problems.

## Data availability statement

The raw data supporting the conclusions of this article will be made available by the authors, without undue reservation.

## Ethics statement

The studies involving humans were approved by Ethics Committee for Clinical Studies of the Timisoara County Emergency Clinical Hospital (registration number 400/29.06.2023). The studies were conducted in accordance with the local legislation and institutional requirements. The participants provided their written informed consent to participate in this study. Written informed consent was obtained from the individual(s) for the publication of any potentially identifiable images or data included in this article.

## Author contributions

AEG: Conceptualization, Data curation, Formal analysis, Funding acquisition, Investigation, Methodology, Project administration, Resources, Software, Supervision, Validation, Visualization, Writing—original draft, Writing—review & editing. DCJ: Conceptualization, Data curation, Formal analysis, Investigation, Methodology, Resources, Software, Validation, Visualization, Writing—original draft. FP: Conceptualization, Writing—original draft. AGM: Conceptualization, Writing—original draft, Writing—review & editing. AA: Conceptualization, Writing—original draft. AZS: Conceptualization, Writing—original draft. AAG: Conceptualization, Writing—original draft.
